# Interobserver Agreement for Endometrial Cancer Characteristics Evaluated on Biopsy Material

**DOI:** 10.1155/2012/414086

**Published:** 2012-02-02

**Authors:** S. Nofech-Mozes, N. Ismiil, V. Dubé, R. S. Saad, Z. Ghorab, A. Grin, I. Ackerman, M. A. Khalifa

**Affiliations:** ^1^Department of Pathology, Sunnybrook Health Sciences Centre, University of Toronto, Toronto, ON, Canada M4N 3M5; ^2^Division of Radiation Oncology, Odette Cancer Centre, Toronto, ON, Canada M4N 3M5

## Abstract

A shift toward a disease-based therapy designed according to patterns of failure and likelihood of nodal involvement predicted by pathologic determinants has recently led to considering a selective approach to lymphadenectomy for endometrial cancer. Therefore, it became critical to examine reproducibility of diagnosing the key determinants of risk, on preoperative endometrial tissue samples as well as the concordance between preoperative and postresection specimens. Six gynaecologic pathologists assessed 105 consecutive endometrial biopsies originally reported as positive for endometrial cancer for cell type (endometrioid versus nonendometrioid), tumor grade (FIGO 3-tiered and 2-tiered), nuclear grade, and risk category (low risk defined as endometrioid histology, grade 1 + 2 and nuclear grade <3). Interrater agreement levels were substantial for identification of nonendometrioid histology (*κ* = 0.63; SE = 0.025), high tumor grade (*κ* = 0.64; SE = 0.025), and risk category (*κ* = 0.66; SE = 0.025). The overall agreement was fair for nuclear grade (*κ* = 0.21; SE = 0.025). There is agreement amongst pathologists in identifying high-risk pathologic determinants on endometrial cancer biopsies, and these highly correlate with postresection specimens. This is ascertainment prerequisite adaptation of the paradigm shift in surgical staging of patients with endometrial cancer.

## 1. Introduction

Surgery is the primary treatment modality for endometrial cancer. In 1988, the International Federation of Gynecology and Obstetrics (FIGO) recommended surgical staging that includes pelvic and para-aortic lymphadenectomy in all cases of endometrial cancer [1]. Based on the data presented by Creasman et al. the frequency of pelvic lymph node metastases is found in 3%, 9%, and 18% in grade 1, 2, and 3 endometrial cancer and of paraaortic involvement 2%, 5%, and 11% in grade is 1, 2, and 3, respectively [2]. Although surgical staging allows for accurate assessment of the extent of disease that guides adjuvant therapy, recent data suggests that staging may not be universally necessary. A shift toward a disease-based therapy applied according to the likelihood of lymph node metastases [3–5] and patterns of failure which are predicted by pathologic determinants has recently been introduced by Mariani et al. [6]. Accordingly, lymph node dissection can be avoided in low-risk patients whilst it should be considered for all non-low-risk population. In their paper, Mariani et al. define the characteristics of low-risk endometrial cancer as endometrioid histology, FIGO grade 1 and 2 (low grade), less than 50% myoinvasion, primary tumor diameter of less than 2 cm, and no gross evidence of extrauterine disease. All others may benefit from surgical staging. As lymphadenectomy in some women with endometrial cancer may be associated with increased risk of perioperative and postoperative morbidity and rarely mortality [7, 8], it is desirable to identify patients who are at significant risk for extrauterine spread and require complete surgical staging while sparing the majority a more morbid procedure. Two of these four factors identified by Mariani et al., namely, grade and cell type, are assessed and available in preoperative specimens. Without commenting on the clinical aspect of this dispute or on the appropriate approach for accurate assessment of the remaining factors (tumor size and depth of myoinvasion), we identified the need to examine the reproducibility of designating tumor grade and cell type in cancer-positive preoperative biopsies and their concordance with the final pathology. Several previous interobserver reproducibility studies assessed the diagnostic performance of gynecologic pathologist in their assessment of endometrial biopsies. These studies have focused on their agreement on the spectrum of endometrial hyperplasias bordering on FIGO grade 1 endometrioid adenocarcinoma or the histologic dating of cycling endometrium [9–13]. 

This study assessed the diagnostic agreement amongst pathologists with special expertise in gynecologic pathology, on the two histologic features present on preoperative endometrial biopsies harboring malignancy, namely, tumor grade and cell type, the two features that can identify risk stratification for likelihood of lymph node involvement and the concordance between preoperative diagnosis and final pathology.

## 2. Materials and Methods

Diagnostic agreement of a panel of six pathologists was assessed by comparing their interpretations of endometrial tissue samples. All six raters are academic pathologists with a subspecialty focus in gynecologic pathology. Their experience in gynecologic pathology ranged from 2 to 17 years. Four of the six pathologists had formal gynecologic pathology fellowship training. One pathologist was practising in site specific sign out practise for 5 years and one participated in the gynepathology service among other disease sites. Each of the six pathologists assessed 105 consecutive cancer-positive endometrial biopsies accessioned between 2001 and 2006, for cell type, tumor grade, nuclear grade, and risk category (to be defined here in after). Each rater was masked to the original pathology report, the review of the other five raters, and the findings in the subsequent hysterectomy. The cell type was dichotomized to Type I that includes endometrioid and mucinous adenocarcinomas versus Type II (nonendometrioid) which includes serous, clear cell, and carcinosarcomas. The analysis of agreement on tumor grade was assessed using both FIGO 3-tiered grading system and separately using the 2-tiered system that merges FIGO grade 1 and 2 into low grade versus high grade as previously described by Scholten et al. [14]. All Type II tumors were assigned a high tumor grade [15]. Raters were provided with the following definitions for nuclear grade prior to the review: nuclear grade 1 was defined as oval, mildly enlarged nucleus with evenly distributed chromatin, nuclear grade 3 was defined as those with markedly enlarged and pleomorphic nuclei, with coarse chromatin and distinct nucleoli, and nuclear grade 2 was characterized by intermediate features [16, 17]. Because the purpose of this study was to examine the agreement on identifying cases in which lymphadenectomy can be omitted, we also evaluated the risk category. Low risk was defined as Type I, FIGO grade 1 + 2 and nuclear grade <3. All others were defined as high risk.

### 2.1. Analyses of Cases Rated as Atypical Complex Hyperplasia

Cases that were classified as atypical complex hyperplasia by any of the pathologists were classified as low risk based on the considerable likelihood of concurrent low-grade carcinoma observed in subsequent resection as previous reported by a GOG study [18]. Notably, most type II endometrial cancer arises on the background of atrophic endometrium and is estrogen independent.

Interrater agreement levels in each category were analyzed by the Fleiss' multiple-rater Kappa statistics with standard error (SE). The general (conventional) consensus scheme for strength of agreement by *κ* values was used in the evaluation as follows: 0.2–0.4, fair; 0.4–0.6, moderate; 0.6–0.8, substantial; 0.8–1, excellent [19].

The concordance between cell type and the 2-tiered tumor grading system on the endometrial biopsy review for each rater and the final pathology report of the hysterectomy was examined. The hysterectomy specimens were not reviewed as this was beyond the scope of this study. Intraoperative consultation by frozen section was not performed.

Ethics approval for this study was obtained from the Research Ethics Board of Sunnybrook Health Sciences Centre.

## 3. Results

All 6 pathologists completed their review addressing all categories.

### 3.1. Cell Type

The overall interrater agreement level for cell type was substantial *κ* = 0.63; SE = 0.025.

### 3.2. Tumor Grade

Table 1 summarizes the agreement among pathologists using 3-tiered grading system. Interrater agreement levels were substantial for identification of high tumor grade (*κ* = 0.64; SE = 0.025). The overall *κ* was only fair when the 3-tiered FIGO grading system was used (*κ* = 0.36, SE = 0.016) compared to substantial level of agreement when the 2-tiered grading system was used (*κ* = 0.64; SE = 0.025). The difference in level of agreement between the two grading systems (2-tiered versus 3-tiered) is ascribed to poor agreement regarding the grade 2 designation using the 3-tiered system (*κ* = 0.09, SE = 0.025). Another factor interfering with interobserver agreement was different threshold for grade 1 endometrioid adenocarcinoma. In 14 cases, 1 to 4 of the 6 raters classified the lesion as complex hyperplasia with atypia. Therefore, the agreement on grade 1 and complex hyperplasia with atypia was only fair (*κ* = 0.35; SE = 0.025 and *κ* = 0.25; SE = 0.025, resp.).

### 3.3. Nuclear Grade

The overall agreement was fair for nuclear grade (*κ* = 0.21; SE = 0.025) although the agreement on identification of nuclear grade 3 was moderate (*κ* = 0.52; SE = 0.016), better than the overall agreement in this category.

### 3.4. Risk Category

**Figure 1:**
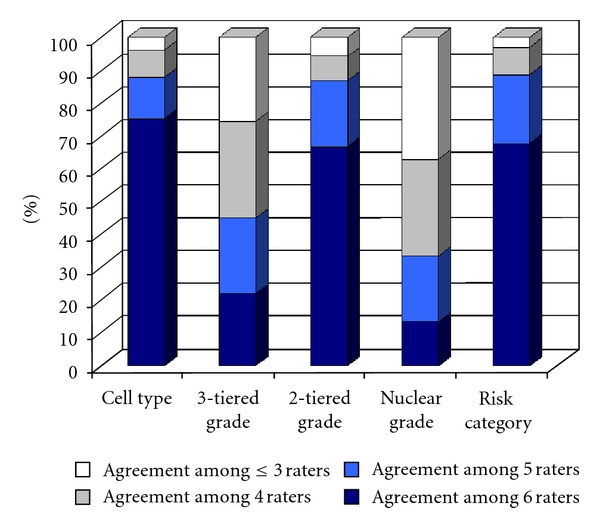
Percentage of cases by degree of agreement in each category.

 When cases were classified into two risk categories with high risk defined by the presence of any of the following features: nonendometrioid cell type, high tumor grade, or high nuclear grade, the interrater agreement level was substantial (*κ* = 0.66; SE = 0.025).  Figure 1 illustrates the proportion of cases by the degree of agreement in each category.

### 3.5. Correlation with Resection Specimens

In 85/105 cases, the primary surgical treatment was carried out in our institution. We examined the concordance between the endometrial biopsy and the final pathology report in the subsequent hysterectomy specimen on cell type and 2-tiered tumor grade for each individual pathologist. On average in 89.2 ± 4.7% of the cases, there was agreement amongst the raters between the cell type determined on the endometrial biopsy with the cell type reported on the resection specimen. Agreement between tumor grade (2-tiered system) determined on the endometrial biopsy and the grade reported in the resection specimens occurred on average in 84.3 ± 5.9% of cases.  Table 2 details the concordance and incidence of under-calling per rater. Overall there were 24 cases with non endometrioid or grade 3 endometrioid adenocarcinoma on final resection. Of those the raters identified 14–18 of them as either nonendometrioid or grade 3.

## 4. Discussion

We examined the level of agreement on the interpretation of preoperative endometrial biopsies regarding cell type, 2-tiered and 3-tiered grading system, nuclear grade, and risk for lymph node involvement categories. To the best of our knowledge, this is the first report on the interobserver reproducibility between multiple pathologists regarding tumor characteristics identified on preoperative endometrial biopsies that are important in selecting patients for complete surgical staging. Interrater agreement levels were substantial for identification of nonendometrioid histology, high tumor grade, and risk category, a combination of factors which determine the need for surgical staging.

In western countries endometrial carcinoma is the most common malignancy of the female genital tract. The extent of surgery, in particular lymphadenectomy, has been the focus of protracted debate in the last two decades [20–29]. Despite FIGO's recommendation for lymphadenectomy, the frequency of lymph node staging varies in different countries and even between different centers. In North America outside the setting of clinical trials and tertiary care centers not all patients with EEA are formally staged [20, 30–35]. Moreover, statistics in both Canada and the United States confirm that, even in countries with a wellrecognized, board-certified subspecialty in gynecologic oncology, only a minority of patients are ever seen by a gynecologic oncologist [36–38]. Previous data has shown that there is a substantial proportion of patients, with a low risk of nodal metastasis and nodal failure following simple surgery who would not benefit from lymphadenectomy and hence can be managed by simple hysterectomy and bilateral salpingo-oophorectomy by general gynecologists in community hospitals while pelvic and paraortic lymphadenectomy should be limited to patients with a significant risk for nodal involvement or nodal failure [39, 40]. In our tertiary care centre, about 55% of all endometrioid endometrial cancer patients are low grade and FIGO stage IA or IB [41]. Based on the Surveillance, Epidemiology, and End Results (SEERs) data summarizing almost 40,000 endometrioid corpus cancers, 63% of cases are stage IA or IB and 83% are low grade [42]. The definition of this high-risk subset varies among studies; however, all include non-endometriod histology and high FIGO grade as important features to identify those who should be surgically staged. These tumor attributes are present in the preoperative specimen and available to the clinician prior to definitive surgery. In a recent review of the literature, Leitao identified that the rate of nodal metastasis reported in previous studies was based on tumor grade and cell type in the final pathology of the resection specimen and not in preoperative biopsy assessment [43]. Given that this information is present preoperatively and can help the clinician decide on the need to undertake lymph node dissection if accurate, it became desirable to examine interobserver agreement on cell type and grade on preoperative specimens as well as their concordance with the final characteristics determined on resection specimens.

When 3-tiered tumor grading system was used, the overall agreement was fair (Table 1). This is attributed to both poor agreement on FIGO grade 2 and on interpreting cases bordering on atypical complex hyperplasia. The distinction between grade 1 and 2 carcinoma is challenging given that it is based on quantitative estimation of nonsquamous solid areas with a cutoff value of 5%. For most pathologists, it is extremely difficult to accurately distinguish between 5% and 6% and even 10%. Moreover, when keratinization is not obvious, some squamous areas may be included in the percentage of solid areas. Our observation regarding poor agreement regarding the designation of FIGO grade 2 is in line with a previous report by Taylor et al. Using a two-tiered system for assessing uterine tumor grade with a delineating value of the presence of 20% nonsquamous solid tumor, the authors found less interobserver variation (*κ* = 0.966) compared to the current three-tiered grading system (*κ* = 0.526) [44]. Similarly, Lax et al. [45] reported superior agreement on the 2-tiered compared with FIGO 3-tiered grading systems. These authors tested the agreement on resection specimens and defined high grade as the presence of at least two of the following three criteria: (1) more than 50% solid growth (without distinction of squamous from nonsquamous epithelium); (2) a diffusely infiltrative, rather than expansive, growth pattern; and (3) tumor cell necrosis. The overall fair *κ* for tumor grade using 3-tiered system (Table 1) also reflects the known disagreement on cases that bordered on atypical complex hyperplasia [46, 47]. Comparison between an artificial 2-tiered FIGO grading and Lax's binary grading system demonstrated that a simple architectural binary grading system that divided tumors into low-grade lesions and high-grade lesions based on the proportion of solid growth (equal or less than 50% or greater than 50%) had superior prognostic power and greater reproducibility [48].

Various reasons for poor reproducibility have been proposed including variably applied criteria for the diagnosis of atypia and complicating features such as metaplasia or polyps. The distinction between atypical complex hyperplasia and FIGO grade 1 endometrioid adenocarcinoma is irrelevant for our discussion as both should be regarded as non-high-risk patients in whom lymphadenectomy could be omitted. As reported earlier by others [49], the overall agreement in our study was fair for nuclear grade although the agreement on identification of nuclear grade 3 was moderate when predefined criteria were provided. In accordance with our observation, poor reproducibility of nuclear grade was documented previously on hysterectomy specimens [50].

It has been argued previously that identification of low-risk patients has been based on the grade assigned on resection and not on biopsy material, and that tumor grade appreciated on biopsies poorly correlates with the final resection specimen [51]. We have shown that preoperative grade and cell type can be identified accurately preoperatively and may be used to assess the risk of lymph node metastases. The likelihood of undercalling that could potentially result in understaging was low. Under-calling occurred in 6–16% of the cases regarding the cell type and in 6–11% of cases regarding tumor grade (Table 1). Seventy percent of the cases undercalled as endometrioid histology on biopsies but proved to be nonendometrioid on hysterectomy specimen had been designated as high-grade endometrioid disease and therefore would not have resulted in undertreatment because they would still be designated to be in the high-risk category requiring staging. Previously reported concordance rates on grade between preoperative and hysterectomy specimens were lower compared with our observation. These studies are based on 3-tiered grading system. The overall concordance reported earlier was 64.5%, and the concordance for grade 3 tumors was significantly higher than that for grade 1 [52]. Another study found that the concordance rates were 20% in grade 1, 61.5% in grade 2, and 77.8% in grade 3 [53]. Eltabbakh et al. found that of those women with grade 1 endometrial carcinoma diagnosed preoperatively, 23% and 6% had grade 2 and 3 disease, respectively, in the hysterectomy specimen [54]. This supports our observation that over 85% of the time high tumor grade determined on biopsy concurred with those of the hysterectomy specimens.

Although all six pathologists involved in this study had a subspecialty focus in gynecologic pathology, they had reasonable diverse background since half of them joined the group less than 6 months prior to the onset of this review. The demonstrated substantial agreement among gynecologic pathologists in identifying cases that require surgical staging needs to be further investigated in the nonacademic setting and among surgical pathologists with no particular gynecologic pathology fellowship training or exceptional expertise.

In conclusion, there is considerable agreement among gynecologic pathologists in identifying high-risk pathologic determinants on the preoperative endometrial cancer biopsies which also correlates with the final pathology. This ascertainment is critical to substantiate the paradigm shift in surgical staging of patients with endometrial cancer.

##  Disclousre

This work will be presented in part to the United States and Canadian Academy of Pathology annual meeting, Boston, MA, March 2009.

## Figures and Tables

**Table 1:** Level of agreement on tumor grade using 3-tiered system.

Diagnosis	*κ*	Strength of agreement [15]
Atypical complex hyperplasia	0.258	fair
FIGO grade 1	0.350	fair
FIGO grade 2	0.096	poor
FIGO grade 3	0.647	substantial
Overall	0.368	fair

**Table 2:** Concordance between interpretation of endometrial biopsies by rater and paired resection specimens (available in 85 patients).

	Cell Type						2-Tiered Tumor Grade					
	Concordant		Discordant		Under-called		Concordant		Discordant		Under-called	
Rater	*N*	%	*N*	%	*N*	%	*N*	%	*N*	%	*N*	%
No. 1	79	92.9	6	7.06	1	1.18	76	89.4	9	10.59	7	8.22
No. 2	74	87.1	10	11.8	5	5.88	72	84.7	13	15.29	8	9.41
No. 3	71	83.5	14	16.5	2	2.35	69	81.2	16	18.82	5	5.88
No. 4	72	84.7	13	15.3	5	5.88	64	75.3	21	24.71	9	10.59
No. 5	79	92.9	6	7.06	2	2.35	71	83.5	14	16.47	7	8.234
No. 6	80	94.1	5	5.88	4	4.71	78	91.8	7	8.235	6	7.06

## References

[1] International Federation of Gynecology and Obstetrics (2007). Corpus cancer staging. *International Journal of Gynecology & Obstetrics*.

[2] Creasman WT, Morrow CP, Bundy BN, Homesley HD, Graham JE, Heller PB (1987). Surgical pathologic spread patterns of endometrial cancer. A gynecologic oncology group study. *Cancer*.

[3] Egle D, Grissemann B, Zeimet AG, Müller-Holzner E, Marth C (2008). Validation of intraoperative risk assessment on frozen section for surgical management of endometrial carcinoma. *Gynecologic Oncology*.

[4] Boronow RC, Morrow CP, Creasman WT (1984). Surgical staging in endometrial cancer: clinical-pathologic findings of a prospective study. *Obstetrics and Gynecology*.

[5] Piver MS, Barlow JJ, Lele SB (1982). Para-aortic lymph node metastasis in FIGO stage I endometrial carcinoma. Value of surgical staging and results of treatment. *New York State Journal of Medicine*.

[6] Mariani A, Dowdy SC, Cliby WA (2008). Prospective assessment of lymphatic dissemination in endometrial cancer: a paradigm shift in surgical staging. *Gynecologic Oncology*.

[7] Hidaka T, Kato K, Yonezawa R (2007). Omission of lymphadenectomy is possible for low-risk corpus cancer. *European Journal of Surgical Oncology*.

[8] Eltabbakh GH, Shamonki J, Mount SL (2005). Surgical stage, final grade, and survival of women with endometrial carcinoma whose preoperative endometrial biopsy shows well-differentiated tumors. *Gynecologic Oncology*.

[9] Sherman ME, Ronnett BM, Ioffe OB (2008). Reproducibility of biopsy diagnoses of endometrial hyperplasia: evidence supporting a simplified classification. *International Journal of Gynecological Pathology*.

[10] Myers ER, Silva S, Barnhart K (2004). Interobserver and intraobserver variability in the histological dating of the endometrium in fertile and infertile women. *Fertility and Sterility*.

[11] Taylor RR, Zeller J, Lieberman RW, O’Connor DM (1999). An analysis of two versus three grades for endometrial carcinoma. *Gynecologic Oncology*.

[12] Kendall BS, Ronnett BM, Isacson C (1998). Reproducibility of the diagnosis of endometrial hyperplasia, atypical hyperplasia, and well-differentiated carcinoma. *American Journal of Surgical Pathology*.

[13] Smith S, Hosid S, Scott L (1995). Endometrial biopsy dating: interobserver variation and its impact on clinical practice. *Journal of Reproductive Medicine for the Obstetrician and Gynecologist*.

[14] Scholten AN, Smit VTHBM, Beerman H, Van Putten WLJ, Creutzberg CL (2004). Prognostic significance and interobserver variability of histologic grading systems for endometrial carcinoma. *Cancer*.

[15] Crum C, Duska LLKMG, Crum C, Lee KR (2006). Adenocarcinoma, carcinosarcoma and other epithelial tumors of the endometrium. *Diagnostic Gynecologic and Obstetric Pathology*.

[16] Ronnett BM, Zaino R, Ellenson L, Kurman RJ, Kurman RJ (2001). Endometrial carcinoma. *Blaustein's Pathology of the Female Genital Tract*.

[17] Nordström B, Strang P, Lindgren A, Bergström R, Tribukait B (1996). Carcinoma of the endometrium: do the nuclear grade and DNA ploidy provide more prognostic information than do the FIGO and WHO classifications?. *International Journal of Gynecological Pathology*.

[18] Trimble CL, Kauderer J, Zaino R (2006). Concurrent endometrial carcinoma in women with a biopsy diagnosis of atypical endometrial hyperplasia: a gynecologic oncology group study. *Cancer*.

[19] Landis JR, Koch GG (1977). The measurement of observer agreement for categorical data. *Biometrics*.

[20] Selman TJ, Mann CH, Zamora J, Khan KS (2008). A systematic review of tests for lymph node status in primary endometrial cancer. *BMC Women’s Health*.

[21] Mariani A, Dowdy SC, Cliby WA (2008). Prospective assessment of lymphatic dissemination in endometrial cancer: a paradigm shift in surgical staging. *Gynecologic Oncology*.

[22] Leitao MM (2008). Current and future surgical approaches in the management of endometrial carcinoma. *Future Oncology*.

[23] Boronow RC (2008). Endometrial cancer and lymph node surgery: the spins continue—a case for reason. *Gynecologic Oncology*.

[24] Hidaka T, Kato K, Yonezawa R (2007). Omission of lymphadenectomy is possible for low-risk corpus cancer. *European Journal of Surgical Oncology*.

[25] Chan JK, Wu H, Cheung MK, Shin JY, Osann K, Kapp DS (2007). The outcomes of 27,063 women with unstaged endometrioid uterine cancer. *Gynecologic Oncology*.

[26] Scholten AN, Van Putten WLJ, Beerman H (2005). Postoperative radiotherapy for Stage 1 endometrial carcinoma: long-term outcome of the randomized PORTEC trial with central pathology review. *International Journal of Radiation Oncology Biology Physics*.

[27] Mariani A, Webb MJ, Keeney GL, Haddock MG, Calori G, Podratz KC (2000). Low-risk corpus cancer: is lymphadenectomy or radiotherapy necessary?. *American Journal of Obstetrics and Gynecology*.

[28] Mohan DS, Samuels MA, Selim MA (1998). Long-term outcomes of therapeutic pelvic lymphadenectomy for stage I endometrial adenocarcinoma. *Gynecologic Oncology*.

[29] Aalders J, Abeler V, Kolstad P, Onsrud M (1980). Postoperative external irradiation and prognostic parameters in stage I endometrial carcinoma. Clinical and histopathologic study of 540 patients. *Obstetrics and Gynecology*.

[30] Nofech-Mozes S, Ghorab Z, Ismiil N (2008). Endometrial endometrioid adenocarcinoma: a pathologic analysis of 827 consecutive cases. *American Journal of Clinical Pathology*.

[31] Leitao MM (2008). Current and future surgical approaches in the management of endometrial carcinoma. *Future Oncology*.

[32] Kwon JS, Carey MS, Cook EF, Qiu F, Paszat L (2007). Patterns of practice and outcomes in intermediate- and high-risk stage I and II endometrial cancer: a population-based study. *International Journal of Gynecological Cancer*.

[33] Corn BW, Dunton CJ, Carlson JA, Xie Y, Valicenti RK (1997). National trends in the surgical staging of corpus cancer: a pattern-of- practice survey. *Obstetrics and Gynecology*.

[34] Aalders J, Abeler V, Kolstad P, Onsrud M (1980). Postoperative external irradiation and prognostic parameters in stage I endometrial carcinoma. Clinical and histopathologic study of 540 patients. *Obstetrics and Gynecology*.

[35] Chan JK, Wu H, Cheung MK, Shin JY, Osann K, Kapp DS (2007). The outcomes of 27,063 women with unstaged endometrioid uterine cancer. *Gynecologic Oncology*.

[36] Kwon JS, Carey MS, Cook EF, Qiu F, Paszat L (2007). Patterns of practice and outcomes in intermediate- and high-risk stage I and II endometrial cancer: a population-based study. *International Journal of Gynecological Cancer*.

[37] Roland PY, Kelly FJ, Kulwicki CY, Blitzer P, Curcio M, Orr JW (2004). The benefits of a gynecologic oncologist: a pattern of care study for endometrial cancer treatment. *Gynecologic Oncology*.

[38] Kwon JS, Carey MS, Goldie SJ, Kim JJ (2007). Cost-effectiveness analysis of treatment strategies for Stage I and II endometrial cancer. *Journal of Obstetrics and Gynaecology Canada*.

[39] Mariani A, Webb MJ, Keeney GL, Haddock MG, Calori G, Podratz KC (2000). Low-risk corpus cancer: is lymphadenectomy or radiotherapy necessary?. *American Journal of Obstetrics and Gynecology*.

[40] Chan JK, Wu H, Cheung MK, Shin JY, Osann K, Kapp DS (2007). The outcomes of 27,063 women with unstaged endometrioid uterine cancer. *Gynecologic Oncology*.

[41] Nofech-Mozes S, Ghorab Z, Ismiil N (2008). Endometrial endometrioid adenocarcinoma: a pathologic analysis of 827 consecutive cases. *American Journal of Clinical Pathology*.

[42] Chan JK, Wu H, Cheung MK, Shin JY, Osann K, Kapp DS (2007). The outcomes of 27,063 women with unstaged endometrioid uterine cancer. *Gynecologic Oncology*.

[43] Leitao MM (2008). Current and future surgical approaches in the management of endometrial carcinoma. *Future Oncology*.

[44] Taylor RR, Zeller J, Lieberman RW, O’Connor DM (1999). An analysis of two versus three grades for endometrial carcinoma. *Gynecologic Oncology*.

[45] Lax SF, Kurman RJ, Pizer ES, Wu L, Ronnett BM (2000). A binary architectural grading system for uterine endometrial endometrioid carcinoma has superior reproducibility compared with FIGO grading and identifies subsets of advance-stage tumors with favorable and unfavorable prognosis. *American Journal of Surgical Pathology*.

[46] Sherman ME, Ronnett BM, Ioffe OB (2008). Reproducibility of biopsy diagnoses of endometrial hyperplasia: evidence supporting a simplified classification. *International Journal of Gynecological Pathology*.

[47] Kendall BS, Ronnett BM, Isacson C (1998). Reproducibility of the diagnosis of endometrial hyperplasia, atypical hyperplasia, and well-differentiated carcinoma. *American Journal of Surgical Pathology*.

[48] Scholten AN, Smit VTHBM, Beerman H, Van Putten WLJ, Creutzberg CL (2004). Prognostic significance and interobserver variability of histologic grading systems for endometrial carcinoma. *Cancer*.

[49] Lax SF, Kurman RJ, Pizer ES, Wu L, Ronnett BM (2000). A binary architectural grading system for uterine endometrial endometrioid carcinoma has superior reproducibility compared with FIGO grading and identifies subsets of advance-stage tumors with favorable and unfavorable prognosis. *American Journal of Surgical Pathology*.

[50] Sagae S, Saito T, Satoh M (2004). The reproducibility of a binary tumor grading system for uterine endometrial endometrioid carcinoma, compared with FIGO system and nuclear grading. *Oncology*.

[51] Leitao MM (2008). Current and future surgical approaches in the management of endometrial carcinoma. *Future Oncology*.

[52] Mitchard J, Hirschowitz L (2003). Concordance of FIGO grade of endometrial adenocarcinomas in biopsy and hysterectomy specimens. *Histopathology*.

[53] Wang X, Huang Z, Di W, Lin Q (2005). Comparison of D&C and hysterectomy pathologic findings in endometrial cancer patients. *Archives of Gynecology and Obstetrics*.

[54] Eltabbakh GH, Shamonki J, Mount SL (2005). Surgical stage, final grade, and survival of women with endometrial carcinoma whose preoperative endometrial biopsy shows well-differentiated tumors. *Gynecologic Oncology*.

